# Disrupted proprioceptive timing estimates contribute to upper limb motor impairment in chronic stroke

**DOI:** 10.1007/s00221-026-07378-8

**Published:** 2026-08-25

**Authors:** Devin S. Austin, Arianna G. Eimont, Jennifer A. Semrau

**Affiliations:** 1 Graduate Program in Biomechanics and Movement Science (BIOMS), University of Delaware, 100 Discovery Blvd., Tower at STAR, 346, Newark, DE 19713 USA; 2 Department of Kinesiology and Applied Physiology, University of Delaware, 100 Discovery Blvd., Tower at STAR, 346, Newark, DE 19713 USA; 3 Department of Biomedical Engineering, University of Delaware, 100 Discovery Blvd., Tower at STAR, 346, Newark, DE 19713 USA

**Keywords:** Stroke, Robotics, Proprioception, Sensorimotor, Temporal processing

## Abstract

In this task, the participants' (*p* < 0.01) and variability (*p* < 0.01) than controls, indicating impaired proprioceptive timing in a task that did not require active motor output from the tested limb. Larger proprioceptive timing errors were strongly correlated with poor performance on VGR (ρ > 0.57, *p* < 0.01) and APM in individuals with stroke (ρ > 0.49, *p* < 0.01). These results suggest that the estimation of timing for dynamic sensory signals is disrupted in individuals with stroke and likely contributes to dysfunction in coordinated movement and static estimations of limb position.

**Supplementary Information:**

The online version contains supplementary material available at https://doi.org/10.1007/s00221-026-07378-8.

## Introduction

Coordinated, accurate movements require the nervous system to continuously monitor limb position in space using proprioception (Proske and Gandevia 2012; Scott 2012; Sherrington 1926; Wolpert and Ghahramani 2000). Muscle spindles provide proprioceptive feedback by monitoring muscle length and the rate at which that length is changing via Type Ia afferents; providing the nervous system with detailed and ongoing information about limb position (Dimitriou 2022; Dimitriou and Edin 2010; Ribot-Ciscar et al. 1998; Sainburg et al. 1993). The timing of these signals is a critical component of accurate limb estimation during movement. In upper-limb reaching movements, proprioceptive feedback can influence longer-latency corrective responses to mechanical perturbations, highlighting the importance of timely spindle-derived information for adjusting an ongoing movement (Forgaard et al. 2016; Pruszynski and Scott 2012). Because proprioceptive signals must travel through peripheral and central processing before influencing these corrections, they must arrive sufficiently early during movement to modify the reaching trajectory (Macefield and Gandevia 1992; Pruszynski and Scott 2012).

Papaioannou et al. demonstrated that greater background activity in Type Ia afferents preceding movement onset predicts small delays during reach movements, indicating that the magnitude of spindle‑mediated feedback can influence the temporal profile of the motor command (Dimitriou 2022; Mendell and Henneman 1971; Papaioannou and Dimitriou 2021). This relationship demonstrates that the magnitude and timing of proprioceptive information can shape the timing at which motor outputs are generated. Because proprioceptive information must be available sufficiently early to assist in movement preparation and online correction, even small delays in proprioceptive feedback can significantly alter ongoing limb state estimates (Scott 2012). Building on this framework, we propose that delayed or poorly integrated proprioceptive feedback may distort real-time limb state estimates, leading to corrective movements that reflect distorted rather than intact sensory estimates. Clinically, proprioceptive impairment is often described in terms of elevated detection thresholds and increased uncertainty in limb position, but these measures also have a temporal dimension: when proprioceptive signals are integrated more slowly or with reduced fidelity, the internal estimate of limb position may lag behind the actual trajectory, producing delays in detecting when the limb reaches a target. In the present work, we focus on this timing‑related aspect of the limb‑state estimator and examine how it relates to established measures of sensorimotor function in individuals with stroke.

Evidence from individuals with large-fiber sensory neuropathy with profound proprioceptive loss supports this idea (Ghez et al. 1995; Sainburg et al. 1993). These individuals demonstrate severe deficits in multi-joint movement coordination despite having intact motor pathways. Critically, their motor impairments are characterized by poor temporal coordination when attempting to synchronize elbow and shoulder movements to produce spatially accurate hand trajectories (Sainburg et al. 1993). This implies that peripheral deafferentation disrupts central sensorimotor integration, preventing the CNS from accurately estimating multi-joint limb states for coordinated control (Sainburg et al. 1993). The deafferentation literature provides a mechanisitic view of how peripherally disrupted proprioceptive processing can degrade coordinated movement; however, central lesions like those that occur in stroke provide a complementary case where damage to central somatosensory areas can impair proprioceptive processing and timing or the integration of these signals, even with intact peripheral processes (Kato and Izumiyama 2015; Yu et al. 2022). This dissociation between sensory and motor impairments has been highlighted by multiple groups where impairment and recovery of sensory function has been shown to be independent in some individuals with stroke (Ingemanson et al. 2019; Semrau et al. 2015). Disentangling disruptions in motor output from the contributions of disordered sensory signals, particularly for dynamic upper limb movements has proven difficult as the two systems are highly integrated. The relationship between these systems has made it difficult to isolate time-based proprioceptive processing impairments as a distinct contributor to sensorimotor impairment after stroke.

Prior literature has also established that individuals with chronic stroke exhibit delayed corrective responses in obstacle avoidance tasks despite relatively intact motor function (Baniña et al. 2017; Mullick et al. 2021). For accurate corrective responses, people must rapidly detect mismatches between predicted and actual limb state, transform these sensory errors into updated motor commands, and execute an appropriately scaled correction. Because these paradigms require active movement of the more affected limb, often with visual feedback, they are constrained by a fundamental confound: any delay in the corrective response could arise from impaired proprioceptive timing, altered use of visual information, slowed motor planning, or motor impairments within the affected limb. It is therefore unclear whether these impairments are primarily sensory or motor in nature, because a purely motor deficit would be expected to alter the magnitude or quality of the correction itself, whereas the main deficit observed was a delay in initiating a correction (Mullick et al. 2021). This delay suggests that stroke may slow the detection and transmission of sensory prediction errors needed to plan and update corrective responses, even though the correction is appropriately scaled once initiated (Miyawaki et al. 2022).

To address this critical gap and isolate proprioceptive timing from motor confounds, we developed an assessment of proprioceptive timing: the Passive Arm Proprioceptive Assessment. In this task, the robot passively moves the participant’s arm to a target position in a continuous circular motion in the absence of visual feedback.

We measure how quickly and reliably participants report when the limb reaches the target. This allows for the isolation of dynamic sensory information from motor execution deficits in individuals with stroke, while evaluating their ability to perceive moment-to-moment spatiotemporal changes in proprioceptive information. We hypothesized that stroke disrupts the temporal precision with which dynamic proprioceptive signals are encoded and integrated, such that ongoing limb-position information is delayed and with greater variability, and that this distorted proprioceptive timing is a significant contributor to impairments in sensorimotor control after stroke. From this hypothesis, we predicted that individuals with stroke would demonstrate delayed and variable proprioceptive timing estimation when relying solely on ongoing proprioceptive signals compared to controls.

## Methods

### Participant demographics

Twenty-eight individuals with chronic stroke and twenty-eight age-matched controls participated in this cross-sectional study (Table 1). Individuals with stroke were recruited through the University of Delaware Stroke Research Registry and control participants were recruited from the community surrounding the University of Delaware. We applied several exclusion criteria to screen for confounding factors. Participants were excluded if they had prior upper-limb surgery or injury (e.g., shoulder replacement), a history of other neurological conditions affecting motor or sensory function (e.g., Multiple Sclerosis, Parkinson's Disease), peripheral neuropathy or other sensory disorders (e.g., Diabetic Neuropathy), postural instability that would compromise participation for longer than one hour, or evidence of visuospatial neglect (Behavioral Inattention Test score < 130) (Hartman-Maeir and Katz 1995). Inclusion criteria specific to individuals with stroke included a Montreal Cognitive Assessment score > 17 (Chiti and Pantoni 2014), stroke in the chronic phase (> 6 months post-stroke), and intact visual fields as determined by visual field confrontation testing. This study was approved by the University of Delaware Institutional Review Board, and all participants provided informed consent.

**Table 1:** Participant Demographics

	Controls (n = 28)	Individuals with Stroke (n = 28)
Age	66.0 [59.75, 69.5]	66.0 [59.5, 76.25]
Sex	Male: N = 11 Female: N = 17	Male: N = 14 Female: N = 14
Dominant Hand	Left: N = 5 Right: N = 23	Left: N = 4 Right: N = 24
Months Post Stroke	–	94.5 [51.5, 124.5]
FIM	–	125.0 [123.0, 126.0]
FMA	–	60.0 [55.25, 65.0]
Visual Field Cut	–	N = 2
Hemisphere of Stroke	–	Left: N = 15, Right: N = 13
Lesion Location {Cortical, Sub Cortical, Cortical + Subcortical}		{8, 17, 2}
MOCA	–	27.0 [24.75, 28.0]
BIT	–	144.0 [143.75, 146.0]
PPB—More Affected Arm	–	7.5 [2.38, 9.0]
TLT {0,1,2,3} More Affected Arm	–	{25, 1, 0, 0}
Tone Present {None, Shoulder, Elbow, Wrist} More Affected Arm	–	{16, 1, 8, 3}

FIM, Functional Independence Measure; FMA, Fugl-Meyer Assessment; MOCA, Montreal Cognitive Assessment; BIT, Behavioral Inattention Test; PPB, Purdue Peg Board; TLT, Thumb Localizing Test. Median [25th percentile, 75th percentile]. Curly brackets are used to indicate the total number of participants in each category

### Clinical measures

All individuals with stroke underwent several clinical measures administered by a physical therapist with over 10 years of experience working with individuals with stroke. Clinical assessments included the Functional Independence Measure (Keith 1987) to assess functional ability, the Fugl-Meyer Assessment (Gladstone et al. 2002) to assess arm and hand motor function, the Montreal Cognitive Assessment(Chiti and Pantoni 2014) to screen for cognitive function, the Behavioral Inattention Test (Hartman-Maeir and Katz 1995) to assess for visuospatial neglect, the Purdue Pegboard Test (Tiffin and Asher 1948) to assess manual dexterity, the Thumb Localizer Test (Hirayama 1999) to measure upper limb position sense, and the Modified Ashworth Scale (MAS) to assess muscular tone. If an individual had tone affecting their shoulder horizontal abduction/adduction or flexion/extension in their wrist or elbow, then they were given a score of 1 to represent the presence of tone.

### Experimental setup

**Fig. 1:**
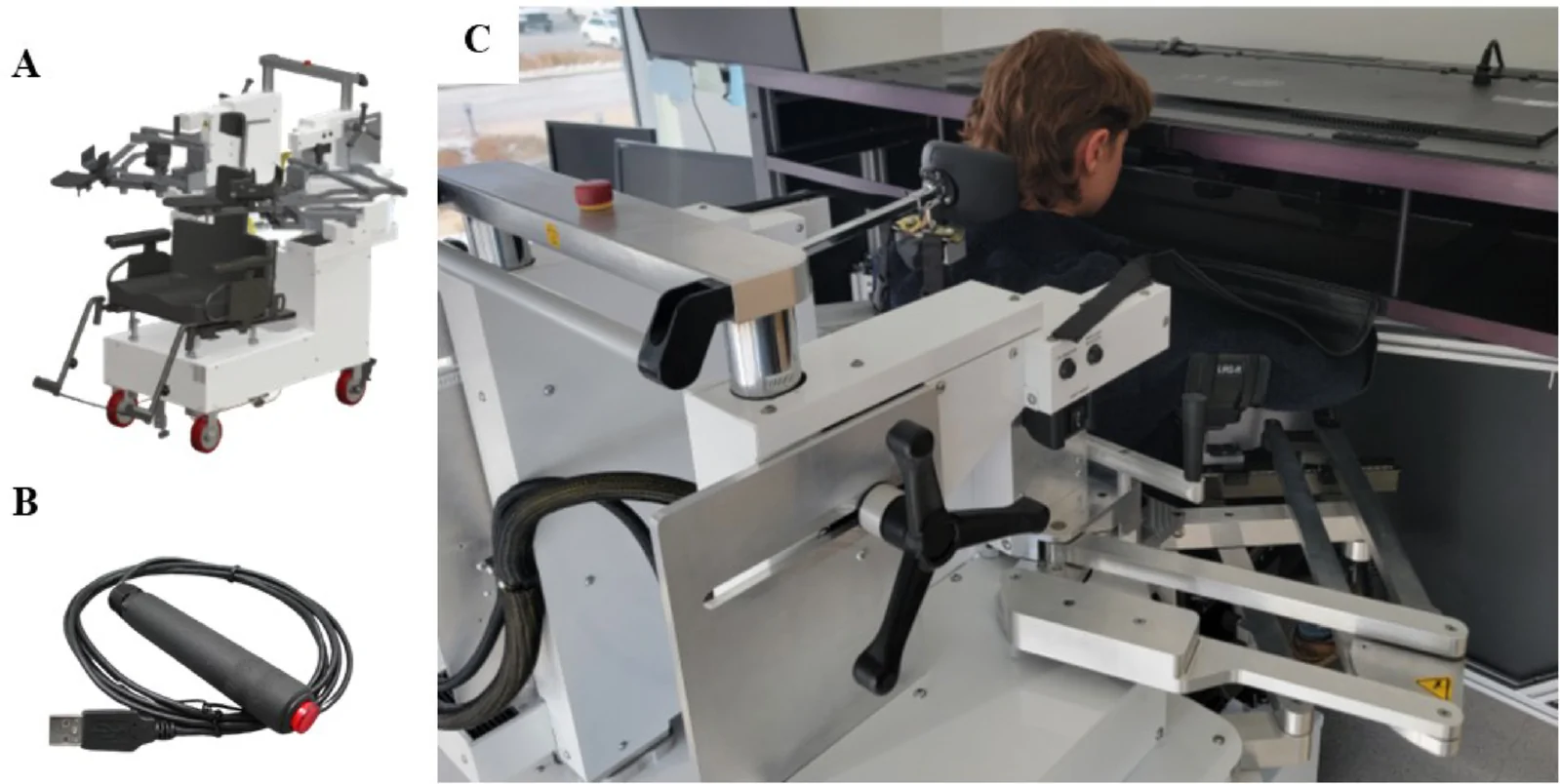
**A** The Kinarm Robotic Exoskeleton captures kinematic data and is used to quantify upper limb sensorimotor behavior. **B** The Delcom Handheld Button used in the Button Timing Task, Temporal Synchronization Assessment (Fast), Temporal Synchronization Assessment (Slow) and the Passive Arm Proprioceptive Assessment. **C** Experimental setup with one participant seated in the Kinarm Robotic Exoskeleton. The hand and arm were occluded by a rigid metal screen while the shoulder and upper arm were occluded by a drape. This is the setup used for all tasks.

All participants completed several tasks using the Kinarm Robotic Exoskeleton (Kinarm, Kingston, ON, Canada) (Fig. 1A) in a single study session lasting approximately 1.5 h. Three of the tasks in the study used a Delcom handheld button (Fig. 1B). Additionally, all individuals with stroke underwent several clinical assessments (described above) lasting approximately one hour. All robotic testing occurred using a Kinarm Robotic Exoskeleton. Arm and forearm segments were supported by elevated platforms which allowed movement in the horizontal plane. During calibration, the more affected arm was positioned in the horizontal plane with the shoulder flexed to ~ 50° and abducted ~ 80°, and the elbow flexed to approximately 90°. The circular path (10 cm radius) of the Passive Arm Proprioceptive Assessment was centered at this workspace location, such that the fingertip moved around a circle lying in the horizontal plane, with movement restricted to shoulder and elbow rotations that maintained the hand near shoulder height. Shoulder abduction was maintained at ~ 80° throughout the experiment. A rigid metal screen occluded the forearm and hand, and a drape occluded the upper arm and shoulder, ensuring that participants could not see any part of the moving limb or infer shoulder position visually. The augmented reality display projected visual targets and task-related text onto the plane of limb movement. Participants therefore saw only the projected targets and a visual cursor when relevant to the task, not their arm. Kinematic data from the robot was filtered with a low pass, 10 Hz cutoff Butterworth filter.

### Novel robotic paradigm: passive arm proprioceptive assessment

**Fig. 2:**
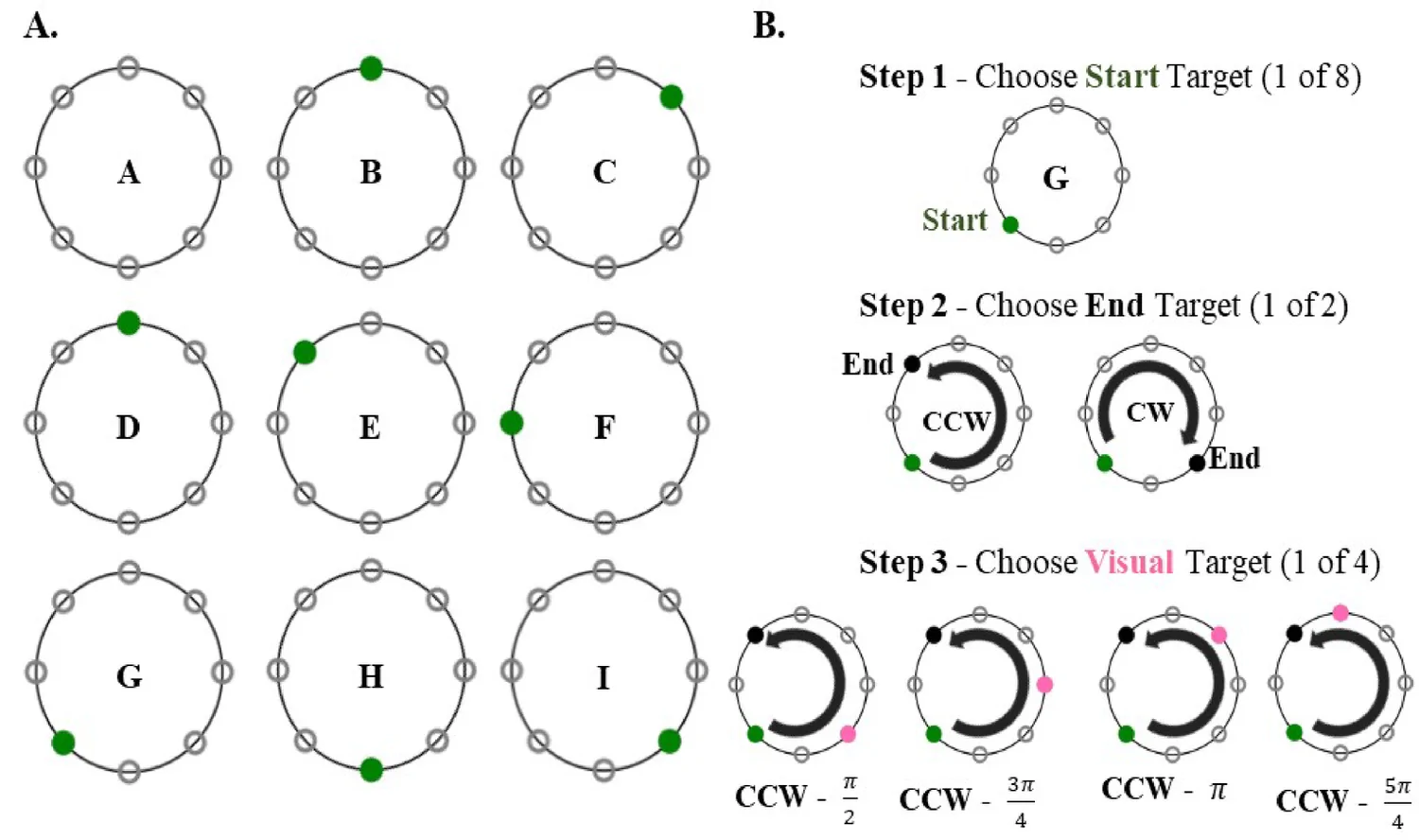
**A** Depiction of target setup and all possible starting target configurations for the Passive Arm Proprioceptive Assessment. Configuration A represents all possible target locations used in the task (noted by the small grey circles on the circular path). Configurations B-I represent all possible Start target locations for the task (start target noted by the green circle). One of these configurations is selected to begin each trial. **B** Depiction of target selection in the *Passive Arm Proprioceptive Assessment.* In this depiction, Configuration G from panel A was pseudorandomly selected as the Start target during Step 1. Start targets indicate the initial position of the limb at the start of a trial. During Step 2, one of two End targets (noted by the black circles) is pseudorandomly selected. End targets indicate the final position where a participant’s limb is moved to. The locations of the two possible End targets are dependent on the chosen Start target, and are always the angle of the Start target ± $\frac{3\pi }{2}$ radians, indicating a clockwise (CW) or counterclockwise (CCW) rotation around the circle. During Step 3, one of four Visual target locations (noted by the pink circles) is pseudorandomly selected, and their locations depend on the previously chosen Start and End targets. Visual targets indicate a single marker that a participant’s hand passes through on the way to the End target. The locations of the four possible Visual targets are always the angle of the Start target + $\frac{\pi }{2}$, $\frac{3\pi }{4}, \pi$ or $\frac{5\pi }{4}$ radians. Each combination of Start target locations, direction of rotation (CCW, CW), and Visual targets produces 64 unique trial combinations that are sampled for each participant. Black arrows represent movement of the hand

**Fig. 3:**
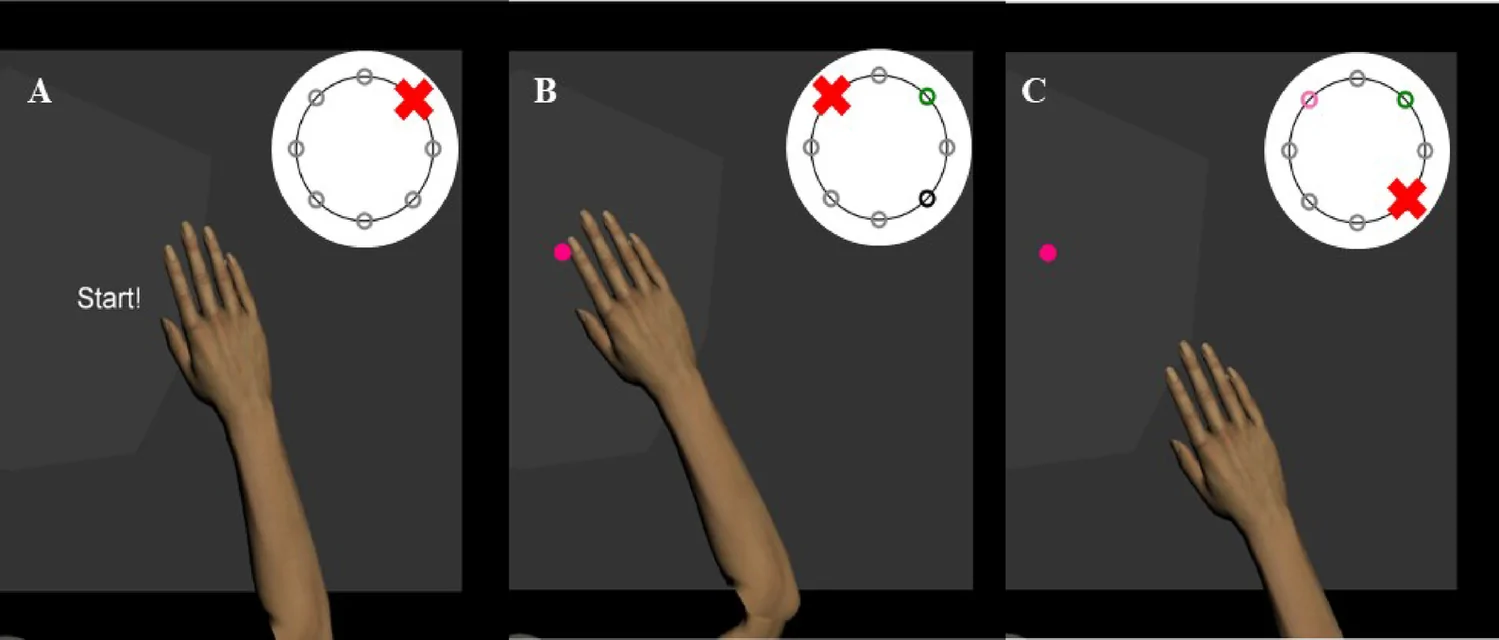
Depiction of a single trial from a control participant performing the Passive Arm Proprioceptive Assessment with their right hand. **A** A participant’s hand was moved to a predetermined start location. That location is the top right marker location demonstrated in the upper right corner of panel A. The red X represents the position of the participants hand on the circular track. **B** The participant’s hand has started moving counterclockwise from the start location and is being passed through a visual target (pink dot). **C** The participant’s hand has continued moving around the circular path and has reached the end target. Throughout the task, participants will only see a “Start” message when beginning a trial and a single visual target. The hand shown in this figure is for demonstration purposes and was not visible to the participant.

To assess an individual’s ability to estimate ongoing limb position and timing while relying solely on proprioceptive information, we developed the Passive Arm Proprioceptive Assessment, in which a participant's unseen limb was passively moved 47.13 cm ($\frac{3\pi }{2}$ radians, 270°) along a circular path with a 10 cm radius and a peak velocity of 20 cm/second. The circular path has eight predetermined start locations spaced $\frac{\pi }{2}$ radians (45°) apart (Fig. 2A). Preceding each trial, a participant’s unseen hand was moved to one of the eight start target locations, and a single pink dot appeared on the screen to represent a visual target (Fig. 2B-Step 1). For individuals with stroke, the more affected limb was passively moved. Participants then saw the text “Start!” on the screen before their fingertip began to move around the circular path, while passing through the visual target, to a pseudorandomly selected end target location based on the participant's hand moving clockwise or counterclockwise. During a trial, participants were instructed to press a handheld button (Fig. 1B) held in their opposite hand when they believe their unseen fingertip passed through the center of the visual target (Fig. 3). The time an individual’s fingertip passed through said target minus the time the participant pressed the button was recorded as their Timing Error, and participants did not receive any visual or performance-based feedback during the task. Individuals with stroke used their less affected limb (determined through clinical assessments of upper limb motor function) when pressing the handheld button while controls used either left or right hand.

To ensure impairments in the Passive Arm Proprioceptive Assessment reflected proprioception specific temporal processing, two control experiments isolated motor reaction time and general temporal perception. The first control experiment utilized a Button Timing Task to assess reaction time when using the handheld button to ensure ipsilesional motor function did not influence reaction times (Semrau et al. 2017). During the task, a single visual target appeared on the screen and participants were instructed to press the button as quickly as possible upon detecting the target. The visual targets were presented on a diamond-shaped grid centered on the screen, with nine possible locations spaced 4 cm apart. Each target was pseudorandomly presented three times, resulting in a total of 27 trials. The interval between trials varied randomly, with a delay of 1000–2000 before a new target appeared. The primary outcome measure for this task was Reaction Time, defined as the interval between the target appearance and the participant’s button press.

The second control experiment involved two versions of a Temporal Synchronization Assessment that assessed whether an individual could appropriately time motor responses to a regularly occurring stimulus, independent of proprioceptive processing. This control experiment allowed us to determine whether any deficits observed in the Passive Arm Proprioceptive Assessment were due to a general difficulty in anticipating and responding to predictable temporal events, rather than a proprioception-specific impairment. In this task, participants observed a single visual target that appeared at fixed time intervals. The target, 2 cm in diameter, was displayed at the center of the screen. Participants were instructed to press a handheld button as soon as the target appeared. The time between the appearance of the target and the button press were recorded. Each version of the task used one of two predetermined intervals between target appearances: 1.57 s (Temporal Synchronization Assessment (Fast)) or 3.14 s (Temporal Synchronization Assessment (Slow)) seconds. These intervals were based on movement times observed in the Passive Arm Proprioceptive Assessment. Participants completed 20 trials per task, and the final 5 trials from each task were used to assess the accuracy of temporal synchronization. If the button was not hit within the specified interval of time, then a trial was added to the end of the task until the participant completed 20 trials.

### Kinarm robotic assessments

**Fig. 4:**
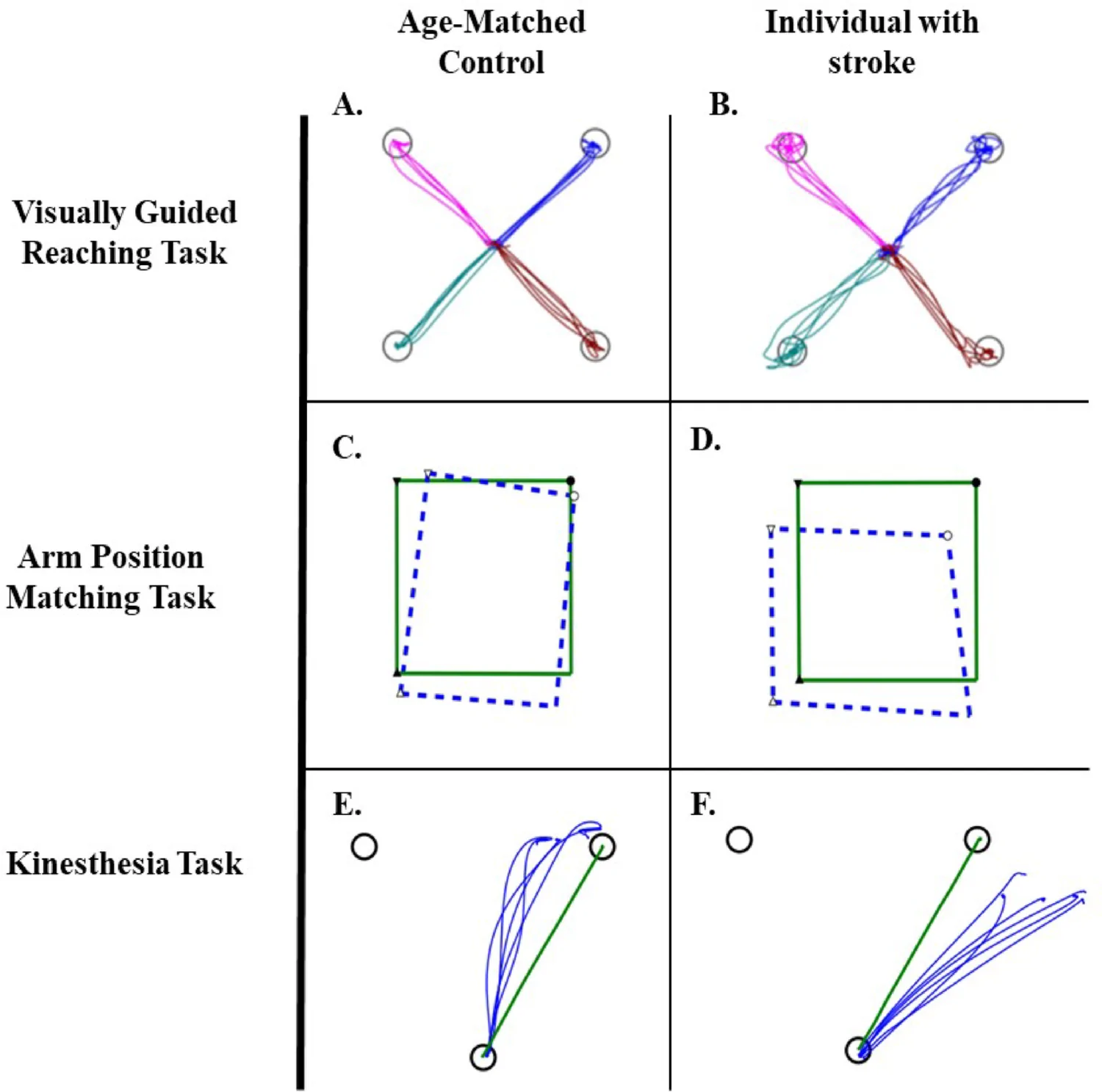
Exemplar movements from a single individual with stroke (right column) and an age-matched control (left column) from three Kinarm Robotic Tasks. **A** Performance from an age-matched control who performed the Visually Guided Reaching task (Task Score: 0.03) and **B** an individual with stroke (Task Score: 2.19). All reaches for the Visually Guided Reaching task are plotted for each of the four target locations. **C** Performance from the Arm Position Matching Task for an age-matched control (Task Score: 0.08) and **D** an individual with stroke (Task Score: 1.05). The solid green lines represent the straight trajectory followed by the robot during passive movement of the arm while the white shapes connected via dotted blue lines represent the average end point locations for the mirror matched limb. **E** Performance to a single target from the Kinesthesia Task for an age-matched control (Task Score: 1.23) and **F** an individual with stroke (Task Score: 0.08). The green line represents the straight trajectory followed by the robot during the passive movement of the arm while the blue lines represent an individual’s movements to the same target.

To determine whether deficits in the temporal precision of proprioception observed in the Passive Arm Proprioceptive Assessment were associated validated behavioral assessments of upper limb sensorimotor function, participants also completed three tasks utilizing the Kinarm Robotic Exoskeleton: The Visually Guided Reaching Task, the Arm Position Matching Task, the Kinesthesia Task (Coderre et al. 2010; Dukelow et al. 2010; Semrau et al. 2013). These tasks have been previously described and utilize a standardized scoring approach. This approach entails generating a single Task Score by normalizing an individual's performance based on age, sex, and handedness; with higher values (Task Scores ≥ 1.96) are generally associated with greater levels of impairment (Scott et al. 2023). Each task score is based on a series of outcomes associated within each task. Brief descriptions of the task and outcomes are provided below:

*Visually Guided Reaching Task (VGR)* Participants used their more-affected arm (arm was counter-balanced in controls) to reaching movements from a central start position to one of four peripheral targets (Coderre et al. 2010) (Fig. 4A, B). A cursor provided real-time visual feedback of their fingertip throughout the movement. This task assesses visuomotor coordination and multi-joint control by quantifying characteristics of the reaching movement such as reaction time, movement speed, initial trajectory, and corrective sub-movements.

*Arm Position Matching Task (APM)* The more-affected arm was passively moved by the robot without visual feedback to one of four equally spaced targets (Dukelow et al. 2010) (Fig. 4C, D). Participants then used their less-affected arm to actively mirror-match the final static position of the more-affected arm and verbally confirmed when they believed they had completed the mirror matched movement. The passive or robotically moved arm was counterbalanced in controls. This task assesses static position sense via end-point matching error and variability across trials.

*Kinesthesia Task (KIN)* The more-affected arm was passively moved by the robot without visual feedback to one of three equally spaced targets (Semrau et al. 2013) (Fig. 4E, F). Participants simultaneously moved their less-affected arm to mirror-match the direction, speed, and timing of the passive movement. They were instructed to begin matching the movement as soon as the more-affected arm started moving. The passive or robotically moved arm was counterbalanced in controls. This task assesses kinesthesia through measures of inter-limb response latency, initial trajectory, and speed between limbs.

### Statistical analyses

Our analyses aimed to (1) assess how chronic stroke impacts the temporal precision of proprioception in a task that relies solely on proprioceptive information and (2) determine how impairments in the temporal precision of proprioception correlate with impairments in motor function (Visually Guided Reaching), and assessments of position sense (APM) and kinesthesia (KIN).

First, we compared performance between individuals with stroke and age-matched controls for the Button Timing Task, Temporal Synchronization Assessment (Fast), Temporal Synchronization Assessment (Slow), and the Passive Arm Proprioceptive Assessment using bootstrapped non-paired permutation tests. Non-paired permutation tests were conducted by resampling each dataset without replacement across 1,000,000 permutations to generate a distribution. For each permutation, a test statistic was computed. The *p*-value was determined by calculating the proportion of permuted test statistics that were as extreme as or more extreme than the observed test statistic in both directions (two-tailed) (Good 2023). To compare performance between groups and across the four different distances in the Passive Arm Proprioceptive Assessment, we performed a mixed-design repeated-measures ANOVA with group (control, individuals with stroke) as a between-subjects factor and four distances as a within-subjects factor on the mean and standard deviation of Timing Error. This analysis tested for main effects of group and distance, as well as a group × distance interaction, to determine whether Timing Error differed between control and stroke participants and whether any group differences were dependent on movement distance. Follow up between-group comparisons were conducted using non-paired permutation tests, because the control and stroke groups comprised independent samples. Within-group comparisons across distances were conducted using paired permutation tests, because the same participants contributed repeated measures across conditions. Permutation tests were used because they do not assume equal variance between groups and are robust to heteroscedasticity. For group comparisons, we also report the Common Language Effect Size (CLES), which represents the probability that a randomly selected value from one group exceeds a randomly selected value from the other group (McGraw & Wong 1992).

Second, we examined the relationships between Timing Error and the Task Scores for VGR, APM, and KIN for individuals with stroke and age matched controls using bootstrapped Spearman correlations. We compared each set of data – with replacement – for one million iterations. We then created a distribution of the *p* values recorded from each iteration and used the mean of this distribution to determine the significance and strength of the relationship. Correlation strength was classified using previously established benchmarks: very weak: ρ < 0.19; weak: 0.20 < ρ < 0.39; moderate: 0.40 < ρ < 0.59; strong: 0.60 < ρ < 0.79; very strong: 0.80 < ρ < 1.00 (Dancey & Reidy, 2007). Values reported with statistical tests are reported as the median [25th percentile, 75th percentile].

## Results

### Participant characteristics

A total of 28 individuals with chronic stroke (66.29 ± 9.78 years old) and 28 age matched neurotypical controls (65.32 ± 7.33 years old) participated in this study, with many participants being right-handed (control N = 23, stroke N = 24). Generally, many of the individuals with stroke had mild or no motor impairment of the upper limb (FMA score 55.18 ± 14.97) and had left hemisphere damage (N = 15). We observed no significant difference in age between individuals with stroke and controls (*p* = 0.69, CLES = 0.47).

### Behavioral differences between controls and individuals with chronic stroke

**Fig. 5:**
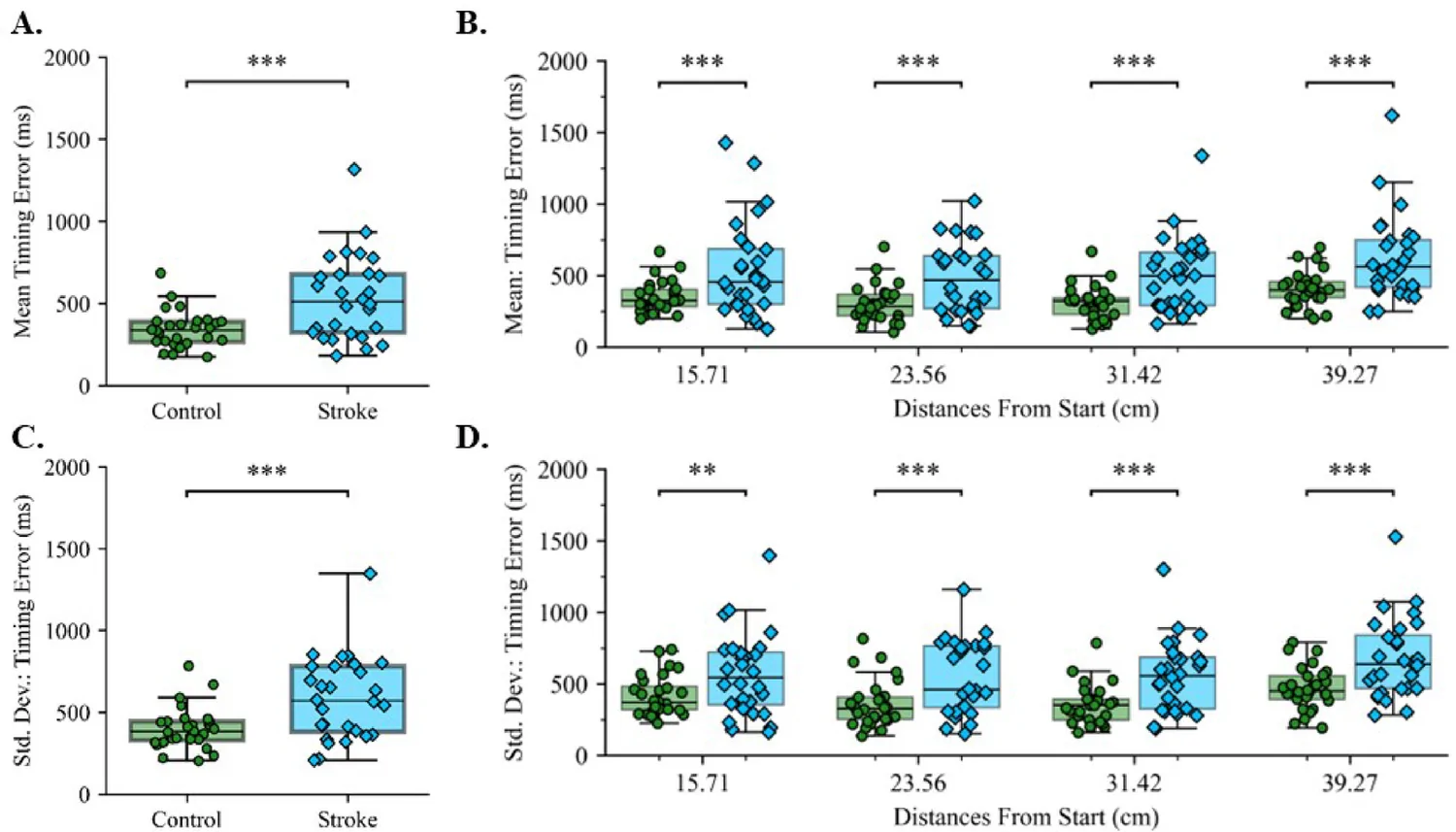
Temporal precision between controls (Green circles) and individuals with stroke (Blue Squares). **A** We observed that individuals with stroke had significantly higher mean Timing Error than controls, **B** and that these differences were observed at each distance from the start target. **C** We also observed that individuals with stroke had significantly higher standard deviation than controls **D** and that this difference persisted across distances. Asterisks indicate significant group differences or distance effects based on non paired permutation tests (*p < 0.05, **p < 0.01, ***p < 0.001)

To examine the impact of chronic stroke on an individual’s ability to localize their limb position during movement when predominantly relying on proprioception, we compared the mean and standard deviation of Timing Error between individuals with stroke and age matched controls across all distances (Fig. 5A, C). We observed that the individuals with stroke – on average – had greater errors (Control: 340 ± 110, Stroke 537 ± 254, *p* < 0.01, CLES = 0.27) and were more variable (Control: 400 ± 126, Stroke 585 ± 247, *p* < 01, CLES = 0.29) when attempting to locate their limb beneath a static visual target during passive limb movement.

Then, we examined how the mean differed between controls and individuals with stroke at each distance from the start target using follow-up paired permutation tests on the significant group × distance interaction from the repeated-measures ANOVA (group: F(1,27) = 6.82, *p* < 0.01; distance: F(3,81) = 14.45, *p* < 0.01; group × distance: F(3,81) = 11.87, *p* < 0.01) (Fig. 5B). In the control group, a majority of pairwise comparisons between distances were statistically significant, indicating that larger Timing Error varied with target distance, with errors being larger at the nearest and farthest distances, with intermediate distances showing smaller errors (Tables 2, 3). In contrast for individuals with stroke, only comparisons involving the farthest distance (39.27 cm) were significantly different, suggesting a reduced or altered distance-dependent scaling of performance relative to controls (Table 3).

**Table 2:** Group Performance at each Distance

Distances:	15.71	23.56	31.42	39.27
Control:	351 ± 109 ms	301 ± 128 ms	308 ± 115 ms	402 ± 121 ms
Stroke:	536 ± 322 ms	484 ± 235 ms	508 ± 251 ms	619 ± 289 ms

**Table 3:** Timing Error Comparisons Across Distances

Distance Comparisons	Controls		Stroke	
	Mean	Standard Dev	Mean	Standard Dev
15.71 vs 23.56	**CLES = 0.65	*CLES = 0.65	CLES = 0.52	CLES = 0.65
15.71 vs 31.42	**CLES = 0.60	**CLES = 0.63	CLES = 0.50	**CLES = 0.63
15.71 vs 39.27	**CLES = 0.36	*CLES = 0.38	CLES = 0.39	**CLES = 0.39
23.56 vs 31.42	CLES = 0.46	CLES = 0.56	CLES = 0.47	CLES = 0.49
23.56 vs 39.27	**CLES = 0.26	**CLES = 0.28	**CLES = 0.37	**CLES = 0.36
31.42 vs 39.27	**CLES = 0.26	**CLES = 0.26	**CLES = 0.38	**CLES = 0.38

^**^p < 0.01, * *p* < 0.05

Furthermore, we examined whether the standard deviation of Timing Error showed a similar group × distance pattern. A repeated-measures ANOVA for the standard deviation of Timing Error revealed significant main effects of group and distance, as well as a significant group × distance interaction (group: F(1,27) = 7.83, *p* < 0.01; distance: F(3,81) = 12.39, *p* < 0.01; group x distance: F(3,81) = 11.46, *p* < 0.01) (Fig. 5D). In the control group, variability generally increased with distance from the start target, with significant pairwise differences for most distance comparisons; however again, 23.56 and 31.42 cm did not significantly differ (*p* = 0.50, CLES = 0.46) (Table 3). This pattern indicates progressively greater between‑trial variability at more distant targets, with similar variability at the two intermediate distances. In contrast, for individuals with stroke, only comparisons involving the farthest distance (39.27 cm) were significantly different, again suggesting that distance‑dependent increases in variability are altered in individuals with stroke relative to controls (Table 3).

### The relationships between robotic assessments with primary outcome measures

**Fig. 6:**
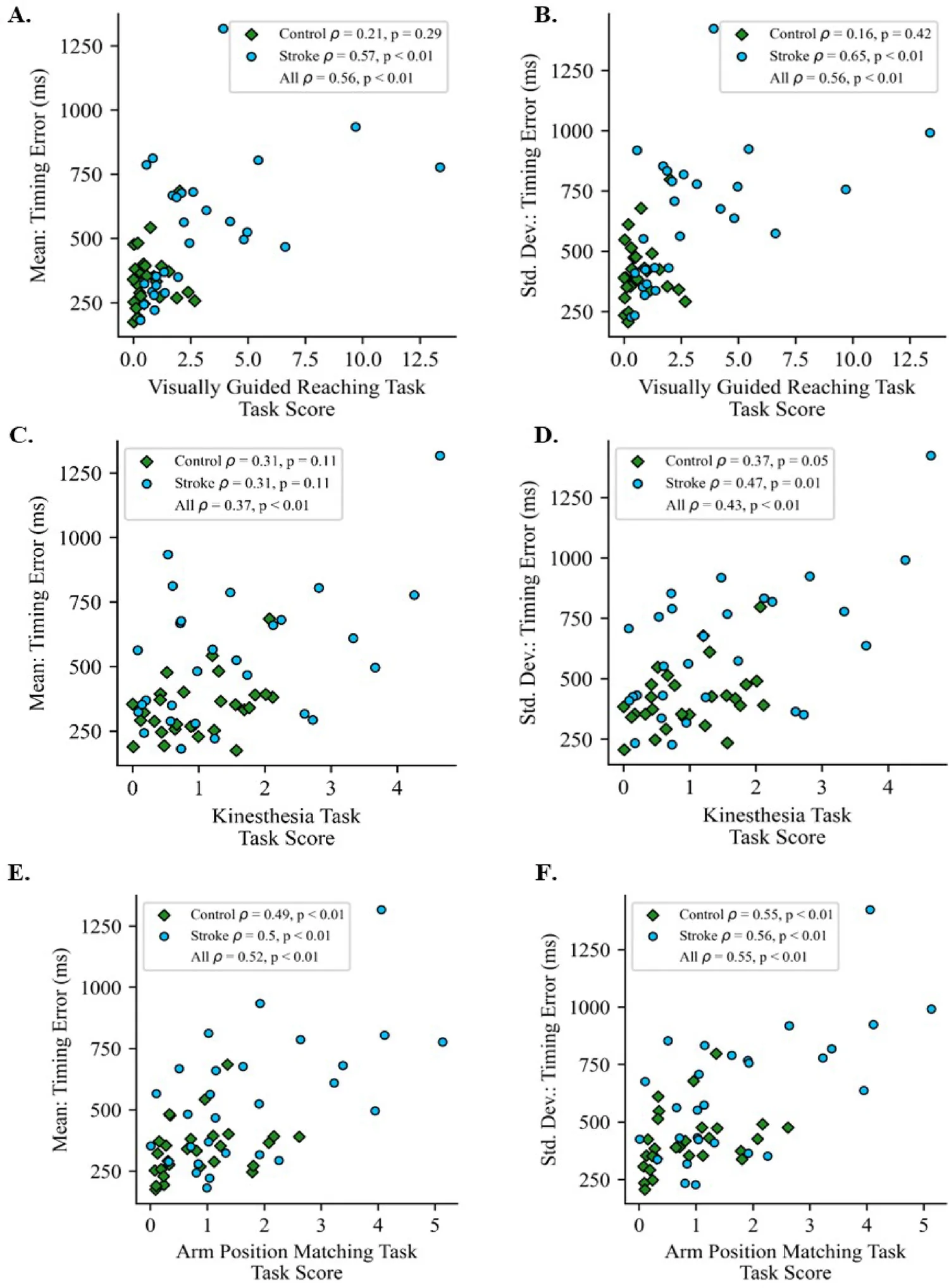
Relationships between temporal precision and Kinarm Robotic Assessments for controls (green circles) and individuals with stroke (blue squares). **A** Mean Timing Error as a function of Visually Guided Reaching Task score. **B** Standard deviation of Timing Error as a function of Visually Guided Reaching Task score. **C** Mean Timing Error as a function of Kinesthesia Task score. **D** Standard deviation of Timing Error as a function of Kinesthesia Task score. **E** Mean Timing Error as a function of Arm Position Matching Task score. **F** Standard deviation of Timing Error as a function of Arm Position Matching Task score. Larger mean and standard deviations of Timing Error were associated with higher Kinarm Task Scores in the Visually Guided Reaching and Arm Position Matching tasks, indicating that individuals with poorer reaching and position sense tended to have greater error when estimating limb position during movement (Spearman ρ and *p* values shown in panel legends).

To determine how delays in proprioceptive timing estimation relate to visually guided reaching, position sense, and kinesthesia, we examined the correlations between mean and standard deviation of Timing Error from the Passive Arm Proprioceptive Assessment and performance on all three Kinarm robotic assessments. For VGR (Fig. 6A, B), we observed no significant relationships between Timing Error and the VGR Task Score in the control group (mean: ρ = 0.21, *p* = 0.29; standard deviation: ρ = 0.16, *p* = 0.42). In contrast, in individuals with stroke, Timing Error showed significant moderate to strong correlations with the VGR Task Score (mean: ρ = 0.57, *p* < 0.01; standard deviation: ρ = 0.65, *p* < 0.01), indicating that participants with larger impairments in proprioceptive timing estimates during a passive, no‑vision task also tended to have larger impairments in visually guided reaching during active movements when visual feedback was present.

For KIN (Fig. 6C, D), Timing Error was not significantly correlated with the KIN Task Score in the control group (mean: ρ = 0.31, *p* = 0.11; standard deviation: ρ = 0.37, *p* = 0.05). In the stroke group, mean Timing Error was not significantly related to the KIN Task Score (mean: ρ = 0.31, *p* = 0.11), whereas the standard deviation of Timing Error showed a moderate, significant correlation with the KIN Task Score (standard deviation: ρ = 0.47, *p* = 0.01). This pattern suggests that trial‑to‑trial consistency in temporal estimations (lower variability in Timing Error) is more closely related to kinesthetic performance than the overall bias in timing (mean Timing Error).

For APM (Fig. 6E, F), Timing Error exhibited moderate significant correlations with the APM Task Score for the control group (mean: ρ = 0.49, *p* < 0.01; standard deviation: ρ = 0.55, *p* < 0.01) and for the stroke group (mean: ρ = 0.50, *p* < 0.01; standard deviation: ρ = 0.60, *p* < 0.01). Together, these findings suggest that impairments in temporal precision are strongly associated with impairments in sensorimotor function.

### Time based control experiments

To determine if the either motor reaction time (Button Timing Task) or perception of time-based responses (Temporal Synchronization Assessment) contributed to the impairments observed in the Passive Arm Proprioceptive Assessment, we examined performance between individuals with stroke and controls. We observed no significant differences between groups for motor reaction time responses on the Button Timing Task (Control: 373 ± 68 ms, Stroke 429 ± 177 ms, *p* = 0.10, CLES = 0.34), or for motor reaction time responses to a regular, time-based stimulus on the Temporal Synchronization Assessment (Fast) (Control: 1524 ± 56 ms, Stroke 1511 ± 198 ms, *p* = 0.95, CLES = 0.43), and the Temporal Synchronization Assessment (Slow) (Control: 3117 ± 48 ms, Stroke 3081 ± 177 ms, *p* = 0.54, CLES = 0.52), indicating both groups had similar reaction times and anticipatory timing at fast and slow temporal patterns.

## Discussion

This study provides new evidence that the timing of dynamic proprioceptive state estimation is impaired after stroke, using a passive assessment designed to substantially reduce motor confounds. By isolating sensory processing from movement execution, our findings show that post‑stroke proprioceptive deficits include not only degraded position sense, but also impaired timing of dynamic sensory information, revealing a temporal component to post‑stroke proprioceptive deficits that may contribute to persistent sensorimotor impairments and/or difficulties in integrating these signals into coordinated control.

### Minimizing confounds to isolate post-stroke proprioceptive deficits

Previous work has consistently demonstrated that proprioceptive deficits are common after stroke, but many studies used paradigms that require active movement of either the more affected or less affected arm, bilateral matching relying on intact interhemispheric transfer, or memory-based judgments inadvertently assessing cognitive load ^34,35,40^. In such designs, weakness, spasticity, or abnormal synergies in either limb can all influence performance, making it unclear whether errors reflect impaired proprioception or limitations in movement execution (Dewald et al. 2001; Hadjiosif et al. 2022b, 2022a). Our Passive Arm Proprioceptive Assessment more directly probes online proprioceptive processing during continuous multi-joint motion without voluntary movement, requiring participants to track the unseen limb’s trajectory and report its location in real time. In the Passive Arm Proprioceptive Assessment, the robot passively moves the more affected limb while participants rely on proprioceptive feedback to judge when the hand passes through a target. As such, this paradigm probes the sensorimotor integration processes that update limb-state estimates during ongoing movement, with minimal influence from voluntary motor planning or execution. By sampling the limb in motion and requiring only a simple button press with the opposite hand to report when the unseen arm reaches the target, the task places minimal demands on the affected limb while directly probing the participant’s ongoing perception of limb position. Furthermore, mirror-matching paradigms also introduce confounds from interhemispheric transfer and coordinate transformations, as one limb reproduces the position of the other. Stroke can disrupt interhemispheric inhibition and signal integration between hemispheres, complicating these processes (Casula et al. 2021; Goble 2010; Li et al. 2015). Because the Passive Arm Proprioceptive Assessment requires only a unilateral, real-time report of target arrival rather than bilateral matching, it provides a more direct measure of proprioceptive timing in the more affected arm and reduces the likelihood that task errors are driven by cross-limb mapping or motor output constraints.

### Disrupted temporal precision of proprioception after stroke

We found that stroke disrupts the temporal precision of proprioceptive processing, leading to increased Timing Error even when motor demands are minimized (Fig. 5A, C). In this context, impairments in Timing Error likely reflect altered online sensory processing and the integration of proprioceptive information over time, rather than limitations in motor function (Cross et al. 2024). At the neural level, the temporal processing of proprioception relies on a distributed sensorimotor network that supports the spatial estimation and timing of limb state estimates, including primary and secondary somatosensory cortices, parietal regions such as the supramarginal gyrus, the insula, and motor and premotor areas that jointly support the estimation of limb position during movement (Ben-Shabat et al. 2015; Chowdhury et al., 2020.; Cross et al. 2024; Delhaye et al. 2018; Limanowski and Blankenburg 2016; Lloyd et al. 2003). Disruptions within this network may degrade the speed or fidelity with which ongoing limb-state estimates are updated, producing delayed responses when participants judge when their limb reaches a target (Chilvers et al. 2024; Kenzie et al. 2023; Ward et al. 2006).

Mechanistically, one possible contributor is a reduction in the fidelity of processing proprioceptive signals within the somatosensory and parietal regions that may prevent up-to-date representations of ongoing limb position (Lacruz et al. 1991; Rocchi et al. 2016; Sweetnam and Brown 2013). A second possible mechanism is the disruption of predictive state estimation supported by sensorimotor networks involving parietal, premotor, and cerebellar systems; leading to inaccurate estimates of limb position (Desmurget et al. 1999; Limanowski and Blankenburg 2016, 2017; Mulliken et al. 2008). These predictive processes generate forward estimates of limb position based on outgoing motor commands and incoming sensory feedback; however, when they are compromised, the system may rely more heavily on noisy/delayed sensory signals, which in turn can increase temporal uncertainty about limb position during ongoing movement (Pelton et al. 2015; van Beers et al. 2002).

Together, these potential mechanisms support a framework for understanding how temporal impairments lead to the proprioceptive errors observed in this study. With impaired proprioceptive processing, each estimate of limb position during an upper limb movement is based on noisy and/or partially outdated information, allowing sensory estimation errors to accumulate over time and distance and creating progressively larger mismatches between the actual proprioceptive signals and perceived limb position (Cameron et al. 2015). This error accumulation in proprioceptive state estimation provides a mechanistic explanation for why Timing Error was greatest at the farthest movement distance in the present task and tended to vary with distance, even though the pattern was not strictly monotonic (Fig. 5B, D). This phenomenon has also been described in the spatial domain, where proprioceptive biases and variability are larger for targets farther from the body, consistent with accumulating sensory noise over longer distances (Wilson et al. 2010). Within this study, in neurologically intact controls, we observed small increases in error over distance. This pattern is consistent with state‑estimation models and prior studies in which longer movements provide more opportunities for sensory noise and prediction error to accumulate, yet intact sensory and predictive mechanisms can mitigate the accumulation of error over time (Berret et al. 2021; Gillan and Bias 2018; Tulimieri and Semrau 2023; Zacksenhouse 2011). In contrast, individuals with stroke show elevated proprioceptive error across distances, and prior work has demonstrated altered scaling of error with movement distance, indicating that their sensory estimates become disproportionately unreliable when longer movement duration or distance is required (Tulimieri and Semrau 2024). When combined with impaired temporal precision and predictive integration, these distance‑dependent effects likely increase Timing Error in individuals with stroke, such that longer movements reveal a compounding deficit in estimating limb position. Together, these findings suggest that degraded position sense and impaired timing reflect different manifestations of a common disruption in limb‑state estimation, where noisy and temporally degraded proprioceptive signals lead to both increased dynamic timing errors and accumulating endpoint errors. This is especially relevant over longer movement distances where uncertainty has more opportunity to build.

### Differential impact of proprioceptive timing errors across motor and proprioceptive assessments

From a state-estimation perspective, accurate limb control depends on the rapid integration of forward predictions based on efference copy in conjunction with incoming and updated proprioceptive signals. When this integration is temporally degraded after stroke, the internal estimate of hand position may become delayed and noisier relative to the true limb trajectory. The timing errors we observed in the Passive Arm Proprioceptive Assessment therefore reflect an impaired limb-state estimator, in which ongoing updates of limb position are based on noisy or partially outdated information. This impaired limb‑state estimator helps explain why larger temporal errors and variability are associated with poorer endpoint accuracy and increased movement corrections in the Visually Guided Reaching and Arm Position Matching tasks. Deficits in the temporal precision of proprioception likely contribute to broader sensorimotor impairments in individuals with stroke, as demonstrated by significant correlations between Timing Error from the Passive Arm Proprioceptive Assessment and Task Scores from the Visually Guided Reaching and Arm Position Matching tasks. The weaker association with the Kinesthesia task suggests that temporal proprioceptive errors are more closely linked to tasks requiring endpoint accuracy and online control than to tasks emphasizing movement detection and initiation.

These findings align with prior evidence that proprioceptive impairment is associated with broader sensorimotor dysfunction after stroke. The Visually Guided Reaching Task Score is composed of metrics such as path length ratio, number of velocity peaks and movement time, all of which can be influenced by the accumulation of proprioceptive errors during movement (Coderre et al. 2010) (Fig. 6A, B). Similarly, the Arm Position Matching Task Score combines endpoint-focused metrics like spatial shift, end point accuracy, and trial-to-trial variability, where accumulating proprioceptive errors provide clear support for impaired matching performance (Dukelow et al. 2010) (Fig. 6C, D). Compounding errors during passive movement would degrade the final limb position estimate, producing systematic spatial shifts away from the target and higher variability across trials as noisy signals fail to converge on a reliable endpoint (Dukelow et al. 2010; Mochizuki et al. 2019; Moore et al. 2024). In contrast, the Kinesthesia Task Score primarily reflects rapid detection and initiation of movement through metrics like initial direction error, response latency, and peak speed ratio, which engage awareness of movement onset and direction more than ongoing position tracking (Kenzie et al. 2016; Semrau et al. 2013) (Fig. 6E, F). Because performance on the Kinesthesia Task likely depends on intact visuospatial attention and working memory, deficits in these domains may obscure or inflate apparent sensory impairments, making it difficult to disentangle cognitive from proprioceptive contributions to task performance. An explicit endpoint metric would be better to reveal such accumulation in the Kinesthesia Task, highlighting why temporal precision deficits correlate more strongly with reaching ability and endpoint-dependent tasks like the Visually Guided Reaching and Arm Position Matching tasks.

### Limitations

One limitation of the present study is the potential influence of muscle tone on passive movement during the Passive Arm Proprioceptive Assessment. Elevated tone can interfere with smooth passive motion, causing the participant’s fingertip to deviate from the circular track and fail to pass beneath the target. Although several participants in this study exhibited some degree of tone, their data was inspected on a case-by-case basis to ensure that spasticity did not disturb the passive movement path or displace the hand outside the track. Future studies employing passive movement paradigms should carefully account for the presence and degree of tone within their sample and consider its potential impact on task performance. Additionally, stroke-related deficits in general temporal perception could theoretically affect timing judgments when estimating one’s limb location during movement; however, our control studies confirmed that group differences were specific to dynamic proprioceptive processing rather than generalized temporalestimation deficits. A further limitation is that our stroke sample exhibited relatively mild motor impairment, with FMA scores clustered near the upper end of the scale. As a result, our findings should be interpreted as most directly applicable to individuals with mild post-stroke motor impairment, and additional work will be needed to determine whether the same pattern extends to individuals with more severe sensorimotor deficits. Importantly, because the present task does not require active movement of the more affected limb, it may be well suited for future studies that include individuals across a broader range of impairment severity.

## Conclusions

This work demonstrates that the timing of proprioceptive state estimation is impaired after stroke, even when motor demands are reduced, and that these timing errors correlate with deficits in motor function and limb position sense. Together, these findings suggest that impaired proprioceptive processing contributes to the decrement of real‑time limb state estimates that support coordinated sensorimotor control after stroke. The Passive Arm Proprioceptive Assessment provides a novel tool for isolating proprioceptive impairments that result from real-time or ongoing timing estimations. By targeting timing-based impairments that directly contribute to impairments in motor performance, such assessments may aid in the targeted rehabilitation of upper limb impairments after stroke.

## Supplementary Information

Below is the link to the electronic supplementary material.

Supplementary Material 1

## Data Availability

The dataset used in this study can be found at the link below: https://figshare.com/s/151b17b5710b876db125

## References

[CR1] Baniña MC, Mullick AA, McFadyen BJ, Levin MF (2017) Upper limb obstacle avoidance behavior in individuals with stroke. Neurorehabil Neural Repair 31(2):133–146. 10.1177/1545968316662527 27542986

[CR2] Ben-Shabat E, Matyas TA, Pell GS, Brodtmann A, Carey LM (2015) The right supramarginal gyrus is important for proprioception in healthy and stroke-affected participants: a functional MRI study. Front Neurol 6:248. 10.3389/fneur.2015.00248 26696951 PMC4668288

[CR3] Berret B, Conessa A, Schweighofer N, Burdet E (2021) Stochastic optimal feedforward-feedback control determines timing and variability of arm movements with or without vision. PLoS Comput Biol 17(6):e1009047. 10.1371/journal.pcbi.1009047 34115757 PMC8221793

[CR4] Cameron BD, de la Malla C, López-Moliner J (2015) Why do movements drift in the dark? Passive versus active mechanisms of error accumulation. J Neurophysiol 114(1):390–399. 10.1152/jn.00032.2015 25925317 PMC4507974

[CR5] Casula EP, Pellicciari MC, Bonnì S, Spanò B, Ponzo V, Salsano I, Giulietti G, Martino Cinnera A, Maiella M, Borghi I, Rocchi L, Bozzali M, Sallustio F, Caltagirone C, Koch G (2021) Evidence for interhemispheric imbalance in stroke patients as revealed by combining transcranial magnetic stimulation and electroencephalography. Hum Brain Mapp 42(5):1343–1358. 10.1002/hbm.25297 33439537 PMC7927297

[CR6] Chilvers M, Low T, Rajashekar D, Dukelow S (2024) White matter disconnection impacts proprioception post-stroke. PLoS ONE 19(9):e0310312. 10.1371/journal.pone.0310312 39264972 PMC11392420

[CR7] Chiti G, Pantoni L (2014) Use of Montreal Cognitive Assessment in patients with stroke. Stroke 45(10):3135–3140. 10.1161/STROKEAHA.114.004590 25116881

[CR8] Chowdhury RH, Glaser JI, Miller LE (2020) Area 2 of primary somatosensory cortex encodes kinematics of the whole arm. Elife 9:e48198. 10.7554/eLife.48198 31971510 PMC6977965

[CR9] Coderre AM, Zeid AA, Dukelow SP, Demmer MJ, Moore KD, Demers MJ, Bretzke H, Herter TM, Glasgow JI, Norman KE, Bagg SD, Scott SH (2010) Assessment of upper-limb sensorimotor function of subacute stroke patients using visually guided reaching. Neurorehabil Neural Repair 24(6):528–541. 10.1177/1545968309356091 20233965

[CR10] Cross KP, Cook DJ, Scott SH (2024) Rapid online corrections for proprioceptive and visual perturbations recruit similar circuits in primary motor cortex. eNeuro. 10.1523/eneuro.0083-23.2024 38238081 PMC10867723

[CR11] Dancey CP, Reidy J (2007) Statistics without Maths for psychology. Pearson education

[CR12] Delhaye BP, Long KH, Bensmaia SJ (2018) Neural basis of touch and proprioception in primate cortex. Compr Physiol 8(4):1575–1602. 10.1002/cphy.c170033 30215864 PMC6330897

[CR13] Desmurget M, Epstein CM, Turner RS, Prablanc C, Alexander GE, Grafton ST (1999) Role of the posterior parietal cortex in updating reaching movements to a visual target. Nat Neurosci 2(6):563–567. 10.1038/9219 10448222

[CR14] Dewald JPA, Sheshadri V, Dawson ML, Beer RF (2001) Upper-limb discoordination in hemiparetic stroke: implications for neurorehabilitation. Top Stroke Rehabil 8(1):1–12. 10.1310/WA7K-NGDF-NHKK-JAGD 14523747

[CR15] *Dexterit-E 3.9 Addendum: kinarm standard tests summary*. (n.d.). 108.

[CR16] Dimitriou M (2022) Human muscle spindles are wired to function as controllable signal-processing devices. Elife 11:e78091. 10.7554/eLife.78091 35829705 PMC9278952

[CR17] Dimitriou M, Edin BB (2010) Human muscle spindles act as forward sensory models. Curr Biol 20(19):1763–1767. 10.1016/j.cub.2010.08.049 20850322

[CR18] Dukelow SP, Herter TM, Moore KD, Demers MJ, Glasgow JI, Bagg SD, Norman KE, Scott SH (2010) Quantitative assessment of limb position sense following stroke. Neurorehabil Neural Repair 24(2):178–187. 10.1177/1545968309345267 19794134

[CR19] Forgaard CJ, Franks IM, Maslovat D, Chua R (2016) Perturbation predictability can influence the long-latency stretch response. PLoS ONE 11(10):e0163854. 10.1371/journal.pone.0163854 27727293 PMC5058553

[CR20] Ghez C, Gordon J, Ghilardi MF (1995) Impairments of reaching movements in patients without proprioception. II. Effects of visual information on accuracy. J Neurophysiol 73(1):361–372. 10.1152/jn.1995.73.1.361 7714578

[CR21] Gillan DJ, Bias RG (2018) Fitting motivation to Fitts’ law: effect of a penalty contingency on controlled movement. In: Proceedings of the human factors and ergonomics society annual meeting, 62(1), 265–269. 10.1177/1541931218621061

[CR22] Gladstone DJ, Danells CJ, Black SE (2002) The Fugl-Meyer Assessment of Motor Recovery after Stroke: a critical review of its measurement properties. Neurorehabil Neural Repair 16(3):232–240. 10.1177/154596802401105171 12234086

[CR23] Goble DJ (2010) Proprioceptive acuity assessment via joint position matching: from basic science to general practice. Phys Ther 90(8):1176–1184. 10.2522/ptj.20090399 20522675

[CR24] Good P (2023) Permutation tests: a practical guide to resampling methods for testing hypotheses. Springer Science & Business Media

[CR25] Hadjiosif AM, Branscheidt M, Anaya MA, Runnalls KD, Keller J, Bastian AJ, Celnik PA, Krakauer JW (2022a) Dissociation between abnormal motor synergies and impaired reaching dexterity after stroke. J Neurophysiol 127(4):856–868. 10.1152/jn.00447.2021 35108107 PMC8957333

[CR26] Hadjiosif AM, Branscheidt M, Anaya MA, Runnalls KD, Keller J, Bastian AJ, Celnik PA, Krakauer JW (2022b) Dissociation between abnormal motor synergies and impaired reaching dexterity after stroke. J Neurophysiol. 10.1152/jn.00447.2021 35108107 PMC8957333

[CR27] Hartman-Maeir A, Katz N (1995) Validity of the Behavioral Inattention Test (BIT): relationships with functional tasks. Am J Occup Ther 49(6):507–516 7645663 10.5014/ajot.49.6.507

[CR28] Hirayama K, Fukutake T, Kawamura M (1999) ‘Thumb localizing test’ for detecting a lesion in the posterior column: medial lemniscal system. J Neurol Sci. 10.1016/s0022-510x(99)00136-7 10500261

[CR29] Ingemanson ML, Rowe JR, Chan V, Wolbrecht ET, Reinkensmeyer DJ, Cramer SC (2019) Somatosensory system integrity explains differences in treatment response after stroke. Neurology. 10.1212/WNL.0000000000007041 30728310 PMC6442007

[CR30] Kato H, Izumiyama M (2015) Impaired motor control due to proprioceptive sensory loss in a patient with cerebral infarction localized to the postcentral gyrus. J Rehabil Med 47(2):187–190 25268114 10.2340/16501977-1900

[CR31] Keith RA (1987) The functional independence measure: a new tool for rehabilitation. Adv Clin Rehabil 2:6–18 3503663

[CR32] Kenzie JM, Semrau JA, Findlater SE, Yu AY, Desai JA, Herter TM, Hill MD, Scott SH, Dukelow SP (2016) Localization of impaired kinesthetic processing post-stroke. Front Human Neurosci. 10.3389/fnhum.2016.00505 PMC5065994 27799902

[CR33] Kenzie JM, Rajashekar D, Goodyear BG, Dukelow SP (2023) Resting state functional connectivity associated with impaired proprioception post‐stroke. Hum Brain Mapp 45(1):e26541. 10.1002/hbm.26541 38053448 PMC10789217

[CR34] Lacruz F, Artieda J, Pastor MA, Obeso JA (1991) The anatomical basis of somaesthetic temporal discrimination in humans. J Neurol Neurosurg Psychiatry 54(12):1077–1081. 10.1136/jnnp.54.12.1077 1783921 PMC1014683

[CR35] Li Y, Wu P, Liang F, Huang W (2015) The microstructural status of the corpus callosum is associated with the degree of motor function and neurological deficit in stroke patients. PLoS ONE 10(4):e0122615. 10.1371/journal.pone.0122615 25875333 PMC4398463

[CR36] Limanowski J, Blankenburg F (2016) Integration of visual and proprioceptive limb position information in human posterior parietal, premotor, and extrastriate cortex. J Neurosci 36(9):2582–2589. 10.1523/JNEUROSCI.3987-15.2016 26937000 PMC6604875

[CR37] Limanowski J, Blankenburg F (2017) Posterior parietal cortex evaluates visuoproprioceptive congruence based on brief visual information. Sci Rep 7(1):16659. 10.1038/s41598-017-16848-7 29192256 PMC5709509

[CR38] Lloyd DM, Shore DI, Spence C, Calvert GA (2003) Multisensory representation of limb position in human premotor cortex. Nat Neurosci 6(1):17–18. 10.1038/nn991 12483217

[CR39] Macefield G, Gandevia SC (1992) Peripheral and central delays in the cortical projections from human truncal muscles: rapid central transmission of proprioceptive input from the hand but not the trunk. Brain 115(1):123–135 1313733 10.1093/brain/115.1.123

[CR40] McGraw KO, Wong SP (1992) A common language effect size statistic. Psychol Bull 111(2):361–365. 10.1037/0033-2909.111.2.361

[CR41] Mendell LM, Henneman E (1971) Terminals of single Ia fibers: location, density, and distribution within a pool of 300 homonymous motoneurons. J Neurophysiol 34(1):171–187. 10.1152/jn.1971.34.1.171 5540577

[CR42] Miyawaki Y, Otani T, Morioka S (2022) Impaired relationship between sense of agency and prediction error due to post-stroke sensorimotor deficits. J Clin Med 11(12):3307. 10.3390/jcm11123307 35743378 PMC9225153

[CR43] Mochizuki G, Centen A, Resnick M, Lowrey C, Dukelow SP, Scott SH (2019) Movement kinematics and proprioception in post-stroke spasticity: assessment using the Kinarm robotic exoskeleton. J Neuroeng Rehabil 16(1):146. 10.1186/s12984-019-0618-5 31753011 PMC6868757

[CR44] Moore RT, Piitz MA, Singh N, Dukelow SP, Cluff T (2024) The independence of impairments in proprioception and visuomotor adaptation after stroke. J Neuroeng Rehabil 21:81. 10.1186/s12984-024-01360-7 38762552 PMC11102216

[CR45] Mullick AA, Baniña MC, Tomita Y, Fung J, Levin MF (2021) Obstacle avoidance and dual-tasking during reaching while standing in patients with mild chronic stroke. Neurorehabil Neural Repair 35(10):915–928. 10.1177/15459683211023190 34455852

[CR46] Mulliken GH, Musallam S, Andersen RA (2008) Forward estimation of movement state in posterior parietal cortex. Proc Natl Acad Sci U S A 105(24):8170–8177. 10.1073/pnas.0802602105 18499800 PMC2448809

[CR47] Papaioannou S, Dimitriou M (2021) Goal-dependent tuning of muscle spindle receptors during movement preparation. Sci Adv 7(9):eabe0401. 10.1126/sciadv.abe0401 33627426 PMC7904268

[CR48] Pelton TA, Wing AM, Fraser D, van Vliet P (2015) Differential effects of parietal and cerebellar stroke in response to object location perturbation. Front Hum Neurosci 9:293. 10.3389/fnhum.2015.00293 26217208 PMC4499699

[CR49] Proske U, Gandevia SC (2012) The proprioceptive senses: their roles in signaling body shape, body position and movement, and muscle force. Physiol Rev 92(4):1651–1697. 10.1152/physrev.00048.2011 23073629

[CR50] Pruszynski JA, Scott SH (2012) Optimal feedback control and the long-latency stretch response. Exp Brain Res 218(3):341–359. 10.1007/s00221-012-3041-8 22370742

[CR51] Ribot-Ciscar E, Rossi-Durand C, Roll J-P (1998) Muscle spindle activity following muscle tendon vibration in man. Neurosci Lett 258(3):147–150. 10.1016/S0304-3940(98)00732-0 9885952

[CR52] Rocchi L, Casula E, Tocco P, Berardelli A, Rothwell J (2016) Somatosensory temporal discrimination threshold involves inhibitory mechanisms in the primary somatosensory area. J Neurosci 36(2):325–335. 10.1523/JNEUROSCI.2008-15.2016 26758826 PMC6602023

[CR53] Sainburg RL, Poizner H, Ghez C (1993) Loss of proprioception produces deficits in interjoint coordination. J Neurophysiol 70(5):2136–2147. 10.1152/jn.1993.70.5.2136 8294975 PMC10710694

[CR54] Scott SH (2012) The computational and neural basis of voluntary motor control and planning. Trends Cogn Sci 16(11):541–549. 10.1016/j.tics.2012.09.008 23031541

[CR55] Scott SH, Lowrey CR, Brown IE, Dukelow SP (2023) Assessment of neurological impairment and recovery using statistical models of neurologically healthy behavior. Neurorehabil Neural Repair 37(6):394–408. 10.1177/15459683221115413 35932111 PMC10315872

[CR56] Semrau JA, Herter TM, Scott SH, Dukelow SP (2013) Robotic identification of kinesthetic deficits after stroke. Stroke 44(12):3414–3421. 10.1161/STROKEAHA.113.002058 24193800

[CR57] Semrau JA, Herter TM, Scott SH, Dukelow SP (2015) Examining differences in patterns of sensory and motor recovery after stroke with robotics. Stroke 46(12):3459–3469. 10.1161/STROKEAHA.115.010750 26542695

[CR58] Semrau JA, Herter TM, Kenzie JM, Findlater SE, Scott SH, Dukelow SP (2017) Robotic characterization of ipsilesional motor function in subacute stroke. Neurorehabil Neural Repair 31(6):571–582. 10.1177/1545968317704903 28443784

[CR59] Sherrington SCS (1926) The integrative action of the nervous system. Yale University Press

[CR60] Sweetnam DA, Brown CE (2013) Stroke induces long-lasting deficits in the temporal fidelity of sensory processing in the somatosensory cortex. J Cereb Blood Flow Metab 33(1):91–96. 10.1038/jcbfm.2012.135 22990417 PMC3597364

[CR61] Tiffin J, Asher EJ (1948) The Purdue pegboard: norms and studies of reliability and validity. J Appl Psychol 32(3):234–247. 10.1037/h0061266 18867059

[CR62] Tulimieri DT, Semrau JA (2023) Aging increases proprioceptive error for a broad range of movement speed and distance estimates in the upper limb. Front Hum Neurosci. 10.3389/fnhum.2023.1217105 37886690 PMC10598783

[CR63] Tulimieri DT, Semrau JA (2024) Impaired proprioception and magnified scaling of proprioceptive error responses in chronic stroke. J Neuroeng Rehabil 21(1):51. 10.1186/s12984-024-01350-9 38594762 PMC11003069

[CR64] van Beers RJ, Baraduc P, Wolpert DM (2002) Role of uncertainty in sensorimotor control. Philos Trans R Soc B Biol Sci 357(1424):1137–1145. 10.1098/rstb.2002.1101 PMC1693018 12217180

[CR65] Ward NS, Brown MM, Thompson AJ, Frackowiak RSJ (2006) Longitudinal changes in cerebral response to proprioceptive input in individual patients after stroke: an fMRI study. Neurorehabil Neural Repair 20(3):398–405. 10.1177/1545968306286322 16885426 PMC3718227

[CR66] Wilson ET, Wong J, Gribble PL (2010) Mapping proprioception across a 2D horizontal workspace. PLoS ONE 5(7):e11851. 10.1371/journal.pone.0011851 20686612 PMC2912297

[CR67] Wolpert DM, Ghahramani Z (2000) Computational principles of movement neuroscience. Nat Neurosci 3(11):1212–1217. 10.1038/81497 11127840

[CR68] Yu Y, Chen Y, Lou T, Shen X (2022) Correlation between proprioceptive impairment and motor deficits after stroke: a meta-analysis review. Front Neurol 12:688616. 10.3389/fneur.2021.688616 35095706 PMC8793362

[CR69] Zacksenhouse M (2011) The effect of movement noise on internal estimations: possible implications for neural coding. BMC Neurosci 12(Suppl 1):P381. 10.1186/1471-2202-12-S1-P381

