# Supplementary material for: Disrupted proprioceptive timing estimates contribute to upper limb motor impairment in chronic stroke
Source: Exp Brain Res. 2026 Aug 25;244(9):186. doi: 10.1007/s00221-026-07378-8 (PMC13506565; doi:10.1007/s00221-026-07378-8)
Supplement: Supplementary file 1 [file 221_2026_7378_MOESM1_ESM.pdf]

Participant	Lesion Side	Stroke Type	Lesion Location	Lesioned Areas
Stroke 1	Right	Ischemic	Subcortical	Thalamus, internal capsule
Stroke 2	Right	Hemorrhagic	Cortical+Subcortical	Middle Cerebral Artery territory, basal ganglia
Stroke 3	Left	Ischemic	Subcortical	Basal ganglia, lentiform nucleus, centrum semiovale
Stroke 4	Left	Ischemic	Subcortical	Thalamus
Stroke 5	Right	Hemorrhagic	Cortical	Frontotemporal parenchyma
Stroke 6	Right	Ischemic	Subcortical	Thalamus
Stroke 7	Left	Ischemic	Cortical	Frontal lobe, parietal lobe
Stroke 8	Left	Hemorrhagic	Subcortical	Thalamus
Stroke 9	Right	Ischemic	Subcortical	Pons
Stroke 10	Left	Ischemic	Subcortical	Lentiform nucleus, corona radiata
Stroke 11	Left	Ischemic	Cortical+Subcortical	Basal ganglia, corona radiata, parietal lobe
Stroke 12	Left	Ischemic	Cortical+Subcortical	Middle Cerebral Artery territory, frontal lobe, centrum semiovale
Stroke 13	Right	Ischemic	Subcortical	Thalamus, internal capsule, mamillary body
Stroke 14	Right	Ischemic	Cortical	Parasagittal superior frontal gyrus, precentral gyrus
Stroke 15	Right	Ischemic	Cortical	Cingulate gyrus, medial frontal lobe
Stroke 16	Right	Hemorrhagic	Subcortical	Lentiform nucleus
Stroke 17	Left	Ischemic	Subcortical	Putamen, caudate
Stroke 18	Right	Hemorrhagic	Cortical	Frontoparietal, centrum semiovale
Stroke 19	Left	Ischemic	Subcortical	Pons
Stroke 20	Left	Hemorrhagic	Subcortical	Thalamus, posterior limb of the internal capsule
Stroke 21	Left	Ischemic	Cortical	Frontal lobe, parietal, lobe, insula
Stroke 22	Left	Ischemic	Subcortical	Putamen, caudate
Stroke 23	Right	Ischemic	Subcortical	Pons
Stroke 24	Left	Ischemic	Cortical	Middle Cerebral Artery territory
Stroke 25	Right	Ischemic	Cortical	Frontal lobe, temporal lobe
Stroke 26	Left	Ischemic	Subcortical	Thalamus
Stroke 27	Right	Ischemic	Subcortical	Lentiform nucleus, corona radiata
Stroke 28	Right	Hemorrhagic	Subcortical	Lentiform nucleus
